# Stimulation-induced ectopicity and propagation windows in model damaged axons

**DOI:** 10.1007/s10827-014-0521-9

**Published:** 2014-08-12

**Authors:** Mathieu Lachance, André Longtin, Catherine E. Morris, Na Yu, Béla Joós

**Affiliations:** 1 Département de physique, Cégep de l’Outaouais, 820 de la Gappe, Gatineau, Québec, J8T 7T7 Canada; 2 Ottawa-Carleton Institute for Physics, University of Ottawa, 150 Louis Pasteur, Ottawa, ON K1N 6N5 Canada; 3 Neurosciences, Ottawa Hospital Research Institute, Ottawa, ON K1H 8M5 Canada

**Keywords:** Ectopicity onset, Phase locking, Neuropathic pain, Coupled left-shift (CLS), Nav1.6 acquired channelopathies

## Abstract

Neural tissue injuries render voltage-gated Na^+^ channels (Nav) leaky, thereby altering excitability, disrupting propagation and causing neuropathic pain related ectopic activity. In both recombinant systems and native excitable membranes, membrane damage causes the kinetically-coupled activation and inactivation processes of Nav channels to undergo hyperpolarizing shifts. This damage-intensity dependent change, called coupled left-shift (CLS), yields a persistent or “subthreshold” Nav window conductance. Nodes of Ranvier simulations involving various degrees of mild CLS showed that, as the system’s channel/pump fluxes attempt to re-establish ion homeostasis, the CLS elicits hyperexcitability, subthreshold oscillations and neuropathic type action potential (AP) bursts. CLS-induced intermittent propagation failure was studied in simulations of stimulated axons, but pump contributions were ignored, leaving open an important question: does mild-injury (small CLS values, pumps functioning well) render propagation-competent but still quiescent axons vulnerable to further impairments as the system attempts to cope with its normal excitatory inputs? We probe this incipient diffuse axonal injury scenario using a 10-node myelinated axon model. Fully restabilized nodes with mild damage can, we show, become ectopic signal generators (“ectopic nodes”) because incoming APs stress Na^+^/K^+^ gradients, thereby altering spike thresholds. Comparable changes could contribute to acquired sodium channelopathies as diverse as epileptic phenomena and to the neuropathic amplification of normally benign sensory inputs. Input spike patterns, we found, propagate with good fidelity through an ectopically firing site only when their frequencies exceed the ectopic frequency. This “propagation window” is a robust phenomenon, occurring despite Gaussian noise, large jitter and the presence of several consecutive ectopic nodes.

**Electronic supplementary material:**

The online version of this article (doi:10.1007/s10827-014-0521-9) contains supplementary material, which is available to authorized users.

Whether it originates from trauma, ischemia, degenerative disease, sepsis, chemical insults or other causes, neuronal injuries lead to assorted “acquired sodium channelopathies” (e.g., Novak et al. 2009; Bialer 2012). The resulting sick excitable cells manifest diverse abnormalities such as hypersensitivity (Greer et al. 2012, Kocsis and Devor 2000, Liu et al. 2000a), ectopicity, propagation anomalies (Kajander and Bennett 1992; Sheen and Chung 1993; Tal et al. 1999; Chul et al. 2000, Liu et al. 2000b, c, 2002; Amir et al. 2002; Ma and LaMotte 2007; Greer et al. 2012) and neuropathic pain (Coutaux et al. 2005, Truini and Cruccu 2006, Fazen and Ringkamp 2007; Costigan et al. 2009; Nickel et al. 2012). Recently, it has been emphasized (Morris et al. 2012) that a pathological feature of sick excitable cells with acquired sodium channelopathies is bleb-type damage of the excitable (Nav-bearing) membranes. Vicious cycles involving Nav-leak (Wolf et al. 2001), Na/K pump insufficiency and secondary Ca-excitotoxicity (Schafer et al. 2009) are a known pathological syndrome, and bleb-type (Dinic et al. 2012) damage can be seen as part of that syndrome (Morris et al. 2012), providing a plausible mechanistic explanation for Nav-leak in sick excitable cells (Wang et al. 2009; Phillips et al. 2009; Jiang and Gonen 2012).

Using recombinant Nav1.6 (node of Ranvier Nav isoform), Wang et al. (2009) showed, via Na^+^-dye and voltage clamp experiments, that cellular trauma directly elicits TTX-sensitive Na^+^-loading and that membrane aspiration (which produces bleb damage) progressively and irreversibly causes what now is termed Nav-CLS, or “coupled left-shift” (Boucher et al. 2012). With bleb damage, Nav channel activation and inactivation (availability) undergo irreversible shifts in the hyperpolarizing direction (left-shift). Given the tight kinetic coupling between fast activation and fast inactivation in Nav channels (Conti et al. 1984; Bean 2007), their damage-induced shifts will have the same magnitude *LS* (Wang et al. 2009). (Note that CLS is the name of the phenomenon while *LS* is a variable representing the CLS value and is thus italicized.) Because the steady-state *g*_Na_(*V*) left-shifts, this corresponds to a “Nav-leak”. Thus, for mild CLS, which is what interests us here, the steady-state *g*_Na_(*V*) in the healthy *V*_rest_ range will increase, putting ion homeostasis under stress, with the pumps continually overworked.

Previous node of Ranvier modeling established that when pumps are included in the system, mild CLS alone induces action potential (AP) bursting (Boucher et al. 2012) in conjunction with subthreshold oscillations (STOs) (Yu et al. 2012). Such ectopic activity is a feature of neuropathic pain and is also seen in epileptic discharge (Bialer 2012; Volman et al. 2012). With ion gradients held fixed, a large CLS applied to a single node will trigger ectopic activity or block propagation (Boucher et al. 2012). In those computations, *LS* was tested from zero to 30 mV; as it increased, the damaged node first became hypersensitive, then spontaneously active (ectopic) at increasing frequencies until, above a certain *LS*, the adjacent healthy nodes were unable to follow. Thereafter, only a fraction of the APs was transmitted, and, plotted against *LS*, this fraction showed a sequence of plateaus (ie. phase-lockings), interlaced with narrow ranges of aperiodic behavior. When stimulated, the damaged axon achieved faithful transmission for small CLS but showed phase-locked propagation pattern with failures at higher *LS* values (Boucher et al. 2012). These studies of saltatory conduction explored a wide range of *LS* values (0–30 mV) for fixed Nernst potentials, with constant current for stimulation. Additionally, modeling excitability in the context of white matter trauma, Volman and Ng (2013) incorporated Nav-CLS and found alterations in AP amplitude and propagation speed.

Here we model mildly damaged axons (mild Nav-CLS), the situation for incipient diffuse axonal injury. With Na/K pumps operational in all nodes, Nernst potentials are dynamic during saltatory AP propagation. We find that concentration changes too small to be of consequence in healthy neurons can have qualitatively important effects in damaged nodes.

Additionally, by applying periodic or variously timed AP-like (spike) input stimuli, we examine effects of Nav-CLS on AP propagation fidelity. In the case of peripheral neuropathies, these findings would apply to the early, acute phase of injury and neuropathic pain, before central and peripheral sensitization have time to occur. What abnormalities in spike count and timing are to be expected? What propagated patterns will result, and can they explain the painful amplification of normally benign stimuli? These are the additional research questions considered here.

Two main findings are reported: 1) mildly damaged yet quiescent axons, upon receiving normal AP traffic, are triggered into an ectopic mode; 2) although an ectopically firing site dominates axon behavior, normal AP trains of sufficiently high frequency can propagate through this site with minimal alteration. The first finding could explain some neuropathic pain phenomena. The model is presented in *Methods*. The *Results* begin with ectopicity triggering then show how high frequency spike trains propagate under various conditions.

## Methods

A myelinated axon is modeled as *N* = 10 Hodgkin and Huxley (1952) nodes of Ranvier, each an isopotential compartment with membrane voltage *V*_*i*_ (where *i* = node number), with adjacent nodes coupled by an internodal conductance *κ* (see Online Resource 1). Dynamic concentration-dependent Nernst potentials *E*_Na_ and *E*_K_ were implemented as in Kager et al. (2000) and Boucher et al. (2012). Therefore, the model encompasses six transmembrane currents in total : the three currents from the Hodgkin-Huxley model (Na^+^, K^+^ and unspecific leaks), the current caused by the Na/K pumps (modeled using Michaelis-Menten kinetics) and two specific leak currents (Na^+^ and K^+^) used to stabilize the rest state despite the pump current. The rate at which ion concentrations change is proportional to the nodal surface to volume ratio *r*.

All simulations begin with disconnected nodes. In the damaged model, node 6 (or nodes 5,6,7) is traumatized by applying CLS at *t* = 0. Nodes are then connected together at *t* = *t*_*κ*_; this ensures that the intact portion of the axon reaches its steady state before suffering the influence of the damaged node(s), which may or may not reach quiescence. Stimulation at node 1, if present, begins at *t* = *t*_stim_ (see Online Resource 1). This ensures that the damaged node, if it fires periodically, has time to dominate the entire axon before external stimulation begins. Stimulation is applied at precise times, periodically or not, in the form of delta functions, each causing a discontinuous Δ*V*_stim_ change of *V*_1_. In the intact (control) model, each delta function stimulus causes the first node to fire an action potential (AP) and each AP fired by that node propagates faithfully (1:1) to all other nodes, up to a “maximum 1:1 propagation frequency” *f*_max_. Additional details on model design and implementation in C language are given in the *Additional Methods* section of Online Resource 2, including explanations for choices of *N*, Δ*V*_stim_ and *κ* values.

## Results

### Ectopic activity can be triggered by stimulation

**Fig. 1:**
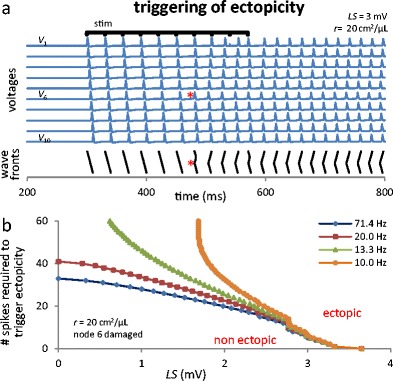
Triggering of ectopic activity. (**a**) Mildly damaged (*LS* = 3 mV) node 6 remains non ectopic if 5 APs or less are propagated, but becomes subtly but distinctly ectopic otherwise (asterisks); note the wave fronts shape change. Stimulation is applied 10 times, every 30 ms, starting at *t*_stim_ = 300 ms. Wave fronts are drawn by connecting maxima of corresponding APs in adjacent nodes. (**b**) Minimal number of APs required to trigger ectopicity. Curves are notably *f*_stim_-dependent only for low *f*_stim_ and large number of APs (because Na/K pump action between spikes is then consequential)

Consider an axon, unstimulated at first, including a single mildly damaged node 6 (0 < *LS* < 3.5 mV), all simulations beginning with *E*_Na_, *E*_K_, *V*, *m*, *h* and *n* at their *LS* = 0 rest state. For this *LS* range, no ectopic activity occurs, the CLS-induced Nav channels leaks being small enough that pumps are able to keep up and the system simply settles to a new rest state (slightly depolarized compared to the healthy axon’s). However, stimulation can *trigger* ectopic behavior, as seen in Fig. 1a for *LS* = 3 mV: apart from a small increase in speed through node 6, the six first APs propagate normally. If stimulation stopped after the fifth AP, the system would return to quiescence, but the sixth AP triggers ectopic behavior (asterisks, Fig. 1a). After this onset, retropropagated APs initiated in node 6 collide with “normal” incoming APs and cancel them. This can be seen on Fig. 1a by following the AP generated by the last stimulus, at *t* = 570 ms : it dies at node 3, along with a retropropagated AP initiated at node 6. This behaviour results in complex wave fronts in the first half of the axon and complete domination of the damaged node over the second half. When stimulation ends, ectopic activity persists until dozens of APs have fired and gradient rundown renders the system unexcitable (*t* ≈ 2000 ms). Na/K pumps then slowly bring it back to its initial quiescent state. This triggered ectopicity is a novel phenomenon, as ectopicity was previously thought to appear immediately when an axon is damaged (Roza et al. 2003).

This onset of ectopicity can be understood from the fact that, at a sufficiently high propagation frequency, APs deplete ionic gradients faster than Na/K pumps can restore them, so all nodes run down at similar rates (see *E*_Na_ and *E*_K_ on Fig. S1 in Online Resource 2). Further, with a diminished initial *E*_K_ (Fig. S2 in Online Resource 2), the system shows periodic ectopic firing *with no* stimulation (Yu et al. 2012). (This is not surprising since removing the K^+^ currents in a membrane at rest leaves the Na^+^ currents out of balance; see also Figs S2 and S3 in Online Resource 2.)

For an intact postsynaptic neuron, the consequence of stimulation-triggered ectopicity in the pre-synaptic neuron would be the arrival of ≈ 90 additional APs, beyond the 6 delivered by an intact pre-synaptic neuron in the illustrated case. This induced ectopic behavior could thus “amplify” a stimulus; occuring in an algoneuron, the brain could interpret a minor stimulus as pain (Fried et al. 2011).

The onset of ectopic activity due to normal AP traffic would take a long time for mildly damaged axons, because the ectopicity-triggering number of spikes (only six in Fig. 1a) would then be high. Fig. 1b shows this number as a function of *LS* for a few stimulation frequencies. The same occurs when three consecutive nodes (5–7) are damaged; the graph is then qualitatively identical to Fig. 1b, apart from a small horizontal compression towards smaller *LS* values (Fig. S3 in Online Resource 2). Ectopicity was detected when the total number of APs reaching node 10 at all *t* > *t*_stim_ was different in the control and damaged systems. The number of spikes propagated is the main determinant for the onset of ectopicity: for all *LS* > 2.5 mV and all *f*_stim_ > 20 Hz, this ectopicity-triggering number of spikes depends on *LS* alone. It appears to also depend on frequency, but only when *f*_stim_ < 20 Hz and *LS* is low, because Na/K pumps then have some time to partially replenish gradients between successive APs.

### High frequency periodic stimulation can drive the ectopic node(s)

**Fig. 2:**
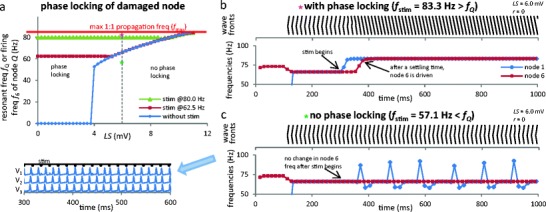
Phase locking of damaged node. (**a**) Steady-state damaged node frequency as function of *LS*. Blue diamonds: ectopic frequency *f*_*Q*_ (without stim); dark red squares and green triangles: frequency *f*_6_ (with stim). Note that *f*_6_ = *f*_stim_ when *f*_*Q*_ < *f*_stim_ but that *f*_6_ = *f*_Q_ when *f*_*Q*_ > *f*_stim_; for *f*_6_ = *f*_stim_, all retropropagation has stopped. Phase locking occurs above the blue (diamonds) curve only. Thus, high frequency AP trains propagate despite the ectopic site. Purple/green stars correspond to (**b**) and (**c**). (**b**) Wave front and time-dependent frequencies *f*_1_ and *f*_6_ when *LS* = 6 mV (*f*_*Q*_ = 66.2 Hz) and *f*_stim_ > *f*_*Q*_. Note the settling time required for *f*_6_ to become equal to *f*_stim_. (**c**) Same as (**b**), with *f*_stim_ < *f*_*Q*_. Note that *f*_6_ never changes after *t*_stim_. *Inset* : nodes 1–3 show the interplay of input APs with retropropagated APs. Note how some of the latter reach node 1 and cause a stimulation failure (absence of AP fired in node 1 at the time of some stimulations), as in Fig. 1a

We now consider axons where the damaged node(s) exhibit(s) spontaneous ectopic firing (3.75 mV < *LS* < 10.75 mV). We will show that propagation can still be achieved by overriding the ectopic site. We first consider simulations where *E*_*Na*_ and *E*_*K*_ are fixed at their healthy values and later expand to dynamic Nernst potentials. (Numerically, this was done by temporarily setting to zero the surface to volume ratio *r*.) Stimulation is periodic unless otherwise specified. Below, *f*_*Q*_ denotes the ectopic firing frequency or “resonant frequency” of damaged node 6 in the unstimulated axon, and *f*_6_ its frequency when stimulated. When *LS* > 3.75 mV (Fig. 2a, blue diamonds curve), ectopic firing occurs spontaneously and frequency *f*_*Q*_ increases monotonically as a function of *LS*.

With no stimulation, the ectopic APs originating in damaged node 6 propagate forward and backward (see both Fig. 2b and c before stimulation begins). With stimulation, *f*_6_ can differ from its unstimulated value *f*_*Q*_, as the two sources of APs (nodes 1 and 6), compete to stimulate the other nodes. When *f*_stim_ > *f*_*Q*_, complex interplays take place in nodes 1–6. Eventually, however, retropropagation stops and 1:1 phase locking occurs with *f*_6_ = *f*_stim_ (Fig. 2a, right part of curves with green triangles and dark red squares; see also Fig. 2b). When *f*_stim_ < *f*_*Q*_, retropropagation never stops and the damaged node dominates the axon’s output. This output, at frequency *f*_*Q*_, shows no *f*_stim_-dependence. Furthermore, no anterograde AP originating in node 1 can reach node 10, since these collide and annihilate with retrograde APs from node 6 (Fig. 2a, left part of curves with green triangles and dark red squares; see also Fig. 2c). Note that 1:1 phase locking becomes unachievable if *f*_*Q*_ exceeds the maximum propagation frequency *f*_max_ of the axon (*LS* > 10.75 mV on Fig. 2a).

When stimulating with *f*_stim_ > *f*_*Q*_, *f*_6_ does not instantaneously switch from *f*_Q_ to *f*_stim_. Rather, a long “settling time” is required, during which APs from node 1 still collide with the retropropagating APs from node 6. Successive collisions occur further down the axon until the collision point reaches the damaged node, which is thenceforth driven at *f*_stim_ (Fig. 2b). Under those conditions, the output frequency at node 10 is *determined by the stimulus at node 1*, as if it were an intact axon, but with a phase shift.

The situation is entirely different when *f*_stim_ < *f*_*Q*_: the ectopic node *fully dominates* the output of the axon (Fig. 2c). The firing frequency of node 6 does not change after stimulation begins, while node 1 fires erratically. This means that APs in nodes 7 to 10 originated in the damaged node and are *completely independent from the stimulus* at node 1.

### Sources of input–output disruption under phase locking conditions

**Fig. 3:**
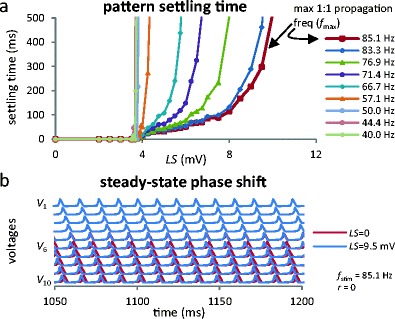
The two effects contributing to *output infidelity* when *f*_stim_ > *f*_*Q*_. (**a**) Settling time increases with *LS* and decreases with *f*_stim_. Note also that for *f*_stim_ < *f*_*Q*_, settling time tends to infinity. Procedures used to generate (**a**) are detailed in the Online Resource 2. (**b**) Voltage traces (after settling time) of intact and damaged (*LS* = 9.5 mV, for which *f*_*Q*_ = 80.4 Hz) axons with same stimulation. Note the phase shift

When *f*_stim_ > *f*_*Q*_, the steady-state frequency is identical to the control system’s but there are, nevertheless, differences between output spike trains in the control and damaged cases. As seen in Fig. 3a, there is a non zero settling time, during which output APs in the control and damaged systems differ. Further, there is a phase shift (Fig. 3b). The combined effect of these two phenomena causes *output infidelity* which we define below.

Figure 3a shows that with increased *LS* and with reduced *f*_stim_, settling times become longer (recall that increasing *LS* elevates *f*_*Q*_). The long settling times are linked to the more robust ectopic firing at such increased *f*_*Q*_. The ectopic node thus generates more retropropagated APs that need to be annihilated by input APs before steady-state 1:1 phase locking is achieved. Further, for a given *LS*, the settling time decreases with increasing *f*_stim_, reaching its minimal value for *f*_stim_ = *f*_max_, the maximum 1:1 propagation frequency (see Fig. 2 and Fig. S1c in Online Resource 2). Thus, the settling time can never be zero, because *f*_stim_ cannot be infinite. During settling time, output spike trains from control and damaged systems can differ significantly.

The phase difference between the outputs of the control and damaged systems (also an increasing function of *LS*) is mostly due to the altered excitability of node 6, resulting in locally accelerated propagation; but it also results from the interplay occurring between nodes 1 and 6. Close inspection reveals that, for the illustrated *LS* value, the damaged node fires *before* the incoming AP reaches it (Fig. 3b). This shows that the phase locking is not a local phenomenon; it involves the interplay of all nodes up to the damaged one.

### A “propagation window” where output infidelity is minimal

A quantitative evaluation of output infidelity requires taking both settling time and phase shift into account. To do so, we generated “output spike trains” (i.e. list of times when maxima of APs occur at node 10) for the damaged and control system fed the same input, and we compared them using the “VP distance” (i.e. the cost-based metric introduced in Victor and Purpura 1996). The VP distance is the *minimal* “cost” of transforming a spike train into another; if adding or removing a spike is assigned an arbitrary unitary cost while shifting a spike by Δ*t* costs *q*Δ*t*. Details and an example are given in Online Resource 2 (see Fig. S4 therein). Note how *q*, an arbitrary parameter with units of cost per ms, weighs the importance given to shifts in spike timings relative to changes in spike count. Physiologically, this *q* parameter could therefore indicate the resolution of a postsynaptic neuron acting as a coincidence detector: when *q*Δ*t* is very low, a spike shifted by Δ*t* could still be detected as coincident with a spike received from another neuron; otherwise, the two spikes would be considered independent. In extreme cases, when *q* = 0, the VP distance equals the difference in spike count, while for large *q*, it equals the sum of both spike counts. This VP distance will hereafter be called “output infidelity”: the higher it is, the more different the two compared spike trains. Note that its use is not restricted to periodic patterns.

**Fig. 4:**
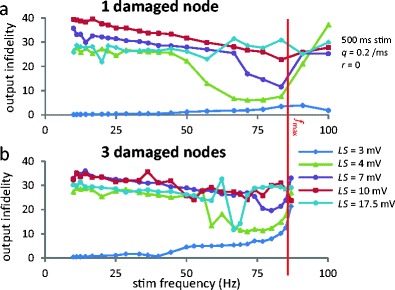
Propagation window. (**a**) Output infidelity as a function of *f*_stim_, evaluated with *q* = 0.2 ms^−1^ for *t* ∈ [310.8, 810.8] ms, i.e. interval between first and last spikes in the control system’s output. Note the wide minimum occurring in a band of large frequencies. This “propagation window” corresponds to *f*_stim_ > *f*_*Q*_ cases. (**b**) Same with CLS affecting nodes 5–7 instead of only node 6. Note that the propagation window still occurs, even though it’s less contrasted. Both panels include non physiologically relevant points (*f*_stim_ > 85.1 Hz) to show that ectopic node(s) dominates the axon again when propagation failure starts occurring

**Fig. 5:**
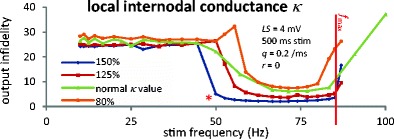
Local increase of internodal conductance reduces infidelity. The *LS* = 4 mV curve from Fig. 4a is called “normal” here; other curves use the same parameters, except *κ*′ (respectively 0.24, 0.375 and 0.45 mS/cm^2^ for the 3 new curves) is used instead of *κ* between nodes 5–6 and 6–7; *κ* remains at 0.3 mS/cm^2^ for all other internodes. See also Fig. S6 in Online Resource 2

**Fig. 6:**
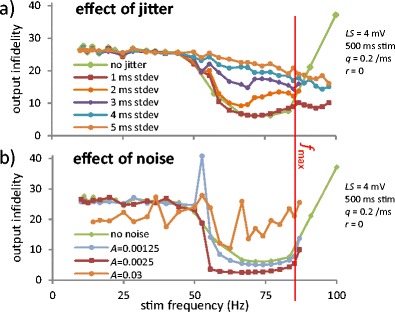
Effects of stochasticity. (**a**) Indidelity as a function of *f*_stim_ for increasing stimulation jitter at node 1. The Gaussian jitter has zero mean and standard deviations as listed. (**b**) Infidelity as a function of *f*_*stim*_ with dynamic current noise modeled as Gaussian white noises added independently in Eq. S1 for all nodes (see Online Resource 2)

The effect of *LS* and *f*_stim_ on output infidelity was investigated. Periodic stimulation was applied for *t* ∈ [300, 800] ms. Since 1.2 ms per internode was allowed for propagation, and there are 9 internodes, output trains were compared for *t* ∈ [310.8, 810.8] ms. Thus, the calculation of output infidelity includes the settling time but not ectopic spikes fired before/after the stimulation period. Figs. 4, 5 and 6 used 500 ms of stimulation and *q* = 0.2 ms^−1^ (see Online Resource 1), and results are independent of these choices (Fig. S5 in Online Resource 2). Fig. 4a plots output infidelity as a function of *f*_stim_. When *LS* = 3 mV (for which no ectopic firing occurs, see Fig. 2a), output infidelity is nearly zero; spike trains propagate faithfully regardless of their frequency. Then over a range of *LS* values (3.75 mV < *LS* < 10.75 mV) and *f*_*stim*_ > *f*_*Q*_ there is still 1:1 phase locking after a settling time (see Figs. 2 and 3). One therefore expects faithful propagation in the interval *f*_*Q*_ < *f*_stim_ < *f*_max_ (Fig. 4a). We define the “propagation window” as the wide and deep minimum of output infidelity that occurs in or around that interval. This window has a different width for each curve in Fig. 4; for example, when *LS* = 4 mV, it begins around *f*_stim_ = 55 Hz (when *f*_stim_ increases past 55 Hz, the output infidelity drops from ≈ 45 to ≈ 6). When *LS* is increased from this value, the propagation window gets narrower (because *f*_*Q*_ increases) and shallower (because the settling time gets longer).

*In vivo* stretched axons would incur damage over several nodes. Fig. 4b, drawn with nodes 5–7 damaged, shows that a propagation window should still be expected in such situations. Note that replicating the damage over three nodes does not triple the infidelity in the propagation window, but barely increases it.

### Increased axoplasmic conductance counteracts ectopicity

An axon traumatized *in vivo* is expected to locally increase or decrease its radius due to immediate response to trauma or to its secondary degeneration (Povlishock and Pettus 1996; Greer et al. 2011; Smith et al. 2013). Since internodal axolemmal conductance is proportional to cross section area, we modeled this radius change by a local *κ* increase or decrease, affecting the internodes located each side of the damaged node. Figure 5 shows results for *LS* = 4 mV (see Fig. S6 in Online Resource 2 for other *LS* values): local *κ* increase widens the propagation window, at least for the mildest damages, while local shrinking leads to opposite results.

These results follow from two mechanisms: 1) the local *κ* increase corresponds to an increased coupling between nodes 5 and 6, which reduces the phase shift described in Fig. 3b; 2) the settling time is also reduced. The latter results from a new ectopic frequency *f*_*Q*_′ which is reduced from *f*_*Q*_, due to the local *κ* increase (see red star on 150 % curve in Fig. 5 with propagation window *f*_stim_ ≥ 50 Hz ≈ *f*_*Q*_′, while Fig. 2a shows *f*_*Q*_ ≈ 53 Hz for the same *LS*). This change in *f*_*Q*_ occurs because a larger part of the charge leaking through Nav channels flows to neighboring nodes, making it harder to reach threshold in the damaged node.

### Propagation window affected oppositely by stimulation jitter and weak dynamic noise

So far, we used deterministic, periodic stimulation. We will now investigate two stochastic effects likely to appear *in vivo*: jitter in the stimulation timing (e.g., due to variability in AP generation upstream of the system’s first node) and noise in the transmembrane currents (e.g., caused by stochastic behavior of the voltage-gated channels). The procedures used to simulate jitter and Gaussian noise are described in the Online Resource 2. Here, *LS* = 4 mV so that curves can be compared with the deterministic result (curve with green triangles in Fig. 4a, reproduced here). Examples of jittered and noisy time series are shown in Fig. S7 in Online Resource 2.

As shown on Fig. 6, the mildest jitter (standard deviation = 1 ms) has a negligible effect on infidelity. However, increasing the jitter further increases infidelity in the propagation window, eventually destroying the window completely. Dynamic noise has richer effects: while strong noise obliterates the propagation window, weak noise (*A* < 0.015; two curves on Fig. 6b) does the opposite: it *improves* the propagation band. Since the output infidelity is reduced in the propagation window, where 1:1 phase-locking already occurs, we infer that the noise reduces the phase shift. This resembles what happens following a local *κ* increase, but the exact mechanism is beyond the scope of this paper. We suspect that this could be a novel instance of “noise-assisted propagation” based on stochastic resonance (Longtin et al. 1991; Ochab-Marcinek et al. 2009), here in a pathological context.

### Ionic gradient depletion gives 1:1 phase locking a finite lifetime

Simulations in the ectopic regime reported in Figs. 2, 3, 4, 5 and 6 were run with *E*_*Na*_ and *E*_*K*_ fixed at healthy values (implemented by using a zero surface to volume ratio, *r*). This simplification allowed for a steady state with a fixed *f*_*Q*_ after a settling time, letting us examine propagation fidelity as a function of parameters characterizing damaged axons. However in reality the surface to volume ratio is non zero. In Fig. S8 from Online Resource 2, the curves shown in Fig. 4a for the output infidelity as a function of *f*_*stim*_ are redrawn with *r* = 20 cm^2^/μL, a high value chosen specifically to illustrate, in a reasonable simulation time, the importance of finite volumes. With this value, a propagation window is observed for short simulations but gradually gets shallower when the simulation is allowed to last longer. This indicates that 1:1 phase locking is lost within a few hundred ms. This loss results from the gradual increase of *f*_*Q*_ associated with the diminishing *E*_K_ values (see Fig. S2 in Online Resource 2), with 1:1 phase locking being lost once the condition, *f*_stim_ > *f*_*Q*_, is no longer respected. This situation can be described by binning the simulation time, computing a time-dependent output infidelity (TDOI) and allowing the presence of a propagation window to be a time-dependent phenomenon. Since nothing stops *f*_*Q*_ from increasing beyond *f*_max_, recurrent transitory propagation windows can be expected in real axons for the duration of time when the Na/K pumps are unable to maintain *E*_K_.

Thus the propagation window found under constant reversal potentials has a finite *r*-dependent lifetime. In other words only a stimulus of finite duration can be transmitted; for ongoing stimulation, the propagation window will appear and disappear via an interplay of stimulus and gradient depletion/recovery time scales.

## Discussion

Nerve damage and its connection to neuropathic pain and its complications are under intense scrutiny. A model of incipient mild nerve injury provides fertile ground to study a host of pain-related phenomena, including allodynia-like amplification of normally benign stimuli. Here, we have studied the interaction of a stimulated node and a one- or three-node damaged zone in a mildly damaged axon.

Damage was modeled as an irreversible coupled left-shift (CLS) of activation and inactivation processes in the Nav channels, a form of Nav-leak that a growing body of evidence links to mechanical or chemical injuries in various types of excitable cells (Sun et al. 2006; Wang et al. 2009; Park et al. 2012; see also Susuki 2013). Depending on the magnitude of *LS*, two behaviors were observed: either the unstimulated system settled to a fixed point or its damaged node immediately exhibited periodic ectopic firing. There were no situations where it started firing ectopically only after a delay.

These two dynamical behaviors underlie our two main findings observed in the presence of pulsatile (AP-like) stimulation. Firstly, we showed that a mildly damaged node of Ranvier, below the range of *LS* values that lead to ectopic firing in the absence of stimulation, can switch behavior and become an ectopic site once the K^+^ concentration gradient is sufficiently depleted (Fig. 1). This resembles an afterdischarge (Coggan et al. 2011) but occurs *before* the end of the stimulus. This result provides a mechanism by which a small stimulus (normally causing 6 APs in the case illustrated on Fig. 1a) triggers ectopicity and causes the axon to fire dozens of APs. The postsynaptic neuron would respond as if the stimulus was much greater than it is in reality. If such a phenomenon occurs in a sensory neuron, it could be responsible for painful amplification of normally benign stimuli, typical of clinically observed conditions like allodynia or hyperalgesia (Costigan et al. 2009).

This result is consistent with observations showing that changes in gene expression are insufficient to cause neuropathic pain, that abberrant Nav activity alone can lead to allodynia, and that blocking Nav channels in animal models is neuroprotective and delays the onset of neuropathic pain (Shankarappa et al. 2012). Our model predicts that the onset of ectopicity can occur not long after the damage, and still occur as long as the neuron lives. Therefore, it can be compared with observations done both before and after central sensitization takes place. In the latter case, it could explain why some patients’ tactile allodynia needs to be set off by scratching or some other stimuli (Ross 2011).

We point out that Fig. S2 shows that the onset of ectopic behaviour can be linked to a change in *E*_K_ alone. Various mechanisms that were left out of our model, such as diffusion or glial K^+^ buffering, are expected to change the rate at which *E*_K_ is diminished upon stimulation, but not the fact that ectopicity is eventually triggered.

Extending the damage to three adjacent nodes shifts the onset of ectopicity towards lower *LS* values (see Fig. S3, an extension of Fig. 1b, in Online Resource 2). It has no other noticeable effect on the phenomenon reported here, which should therefore be expected in experiments despite the method used to damage the axon and the physical extent of the trauma produced (as long as it remains mild enough). We should also note that, according to Fig. 1b, a large decline of *E*_K_ should trigger ectopicity even in an intact axon, as *V*_rest_ depolarizes towards threshold (see also Müller and Somjen 2000; Hao et al. 2013). This occurs sooner in damaged nodes because threshold is hyperpolarized by the CLS-induced window conductance shift. Thus, even though we are dealing with a “sodium leak”, *E*_K_ plays a fundamental role in the excitability dysfunction.

Our second finding concerns the possibility of spike train propagation despite the presence of an ectopic site: while the ectopic node of Ranvier sends APs in both directions in a non-stimulated axon, high frequency stimulation (i.e., higher than the ectopic rate) can enslave the ectopic site by causing it to become phase locked to the incoming signal, a phenomenon similar to behaviors seen in damaged sciatic nerve (Lisney and Devor 1987). Once the short transient period necessary to overcome retropropagating APs passes, the incoming spike train is transmitted with minimal alteration. Note that this occurs in the same way regardless of whether the ectopicity was spontaneous or triggered by stimulation. Interestingly, these results imply that neurons that typically fire at high frequency should be less affected by CLS-type damage. Additionally, this faithful high frequency propagation could mask unhealthy dynamics, emphasising the need to test the frequency dependence of excitability in neurodiagnostic techniques such as threshold tracking (Vucic and Kiernan 2006).

It is important to note that our model as constructed (notably with *r* = 20 cm^2^/μL) will see its gradients depleted when firing occurs over an extended period of time, whether in the healthy (Fig. S1d in Online Resource 2) or damaged state (Fig. S9b in Online Resource 2): as Fig. 1b shows for *LS* = 0, the healthy system becomes ectopic if *f*_stim_ ≥ 20 Hz, revealing sufficient *E*_K_ depletion, but keeps firing continuously with steady state reversal potentials if *f*_stim_ ≤ 13.3 Hz. Our results indicate that the propagation window will eventually disappear because of the depletion of the gradients (this was done here in an exaggerated manner by neglecting homeostatic mechanisms regulating external K^+^ concentration and by choosing a large ratio *r*, as explained in Online Resource 2). Nevertheless a stream of temporally segregated stimuli such as bursts generated at the soma would have a higher likelihood of propagation due the recovery of the gradients during the pauses. One wonders whether there may be a link between these depletion recovery dynamics and the “shooting pain”-type perception of neuropathic stimuli coming in waves (eg. Baron et al. 2010).

This “frequency window” of reliable propagation, where ectopicity is overridden, is a robust phenomenon that should be expected in experiments. Indeed, it still occurs when there are a few consecutive ectopic nodes (Fig. 4b), it resists high levels of jitter in the incoming train (Fig. 6a) and it is even fortified by mild levels of noise in the channel currents (Fig. 6b) through a stochastic resonance effect. In the presence of sustained high frequency input, the 1:1 phase locking of the ectopic site can only be lost if *E*_K_ becomes strongly diminished (Fig. S9 in Online Resource 2). This robust prediction can also lead to a reinterpretation of clinical observations that might have been overlooked. One might conjecture that some behaviors (eg. frenetic scratching or other intense stimulus) practiced by people with tactile allodynia elicit high frequency firing in affected neurons; this firing phase locks 1:1 and produces an expected, and thus normal-feeling, sensation.

We should finally stress that our results are expected to be generally applicable to all neurons. All frequencies and results covered in this article are “scalable” if the model parameters are adjusted. In other words, onset of ectopicity or a propagation window should both be expected in all axons whatever their maximum frequencies are.

## Electronic supplementary material

Below is the link to the electronic supplementary material.

ESM 1 (PDF 70.8 kb)

ESM 2 (PDF 1.63 kb)

ESM 3 (C 181 kb)
