# Supplementary material for: Stimulation-induced ectopicity and propagation windows in model damaged axons
Source: J Comput Neurosci. 2014 Aug 12;37(3):523–31. doi: 10.1007/s10827-014-0521-9 (PMC4224747; doi:10.1007/s10827-014-0521-9)
Supplement: Supplementary file 1 — (PDF 70.8 kb) [file 10827_2014_521_MOESM1_ESM.pdf]

Online Resource 1

Lachance M, Longtin A, Morris CE, Yu N, Joós B.

Table 1: Constant parameters

Symbol	Signification	Normal value	Other value
**Membrane properties**			
C	Nodal capacity	1 μF/cm^2	
g_{Na}	Nav channel maximum conductance	120 mS/cm^2	
g_{K}	Kv channel maximum conductance	36 mS/cm^2	
g_{leak}	Non-specific leak conductance	0.5 mS/cm^2	
$g_{\mathrm{Na,leak}}$	Na-specific leak conductance	0.0625 mS/cm^2	
$g_{\mathrm{K,leak}}$	K-specific leak conductance	0.0205 mS/cm^2	
E_{leak}	Non-specific leak reversal potential	–65.4946 mV	
V_{rest}	Rest potential	–65.4946 mV	
I_{max}	Na/K pumps maximal current	23.6505 μA/cm^2	
$K_{\mathrm{M,Na}}$	Na/K pump Na^+ Michaelis-Menten constant	10 mM	
$K_{\mathrm{M,K}}$	Na/K pump K^+ Michaelis-Menten constant	3.5 mM	
T	Temperature	20 °C	Equation S5 given in (1) for squid axons at 6.3 °C
r	Nodal surface to inner volume ratio	20 cm^2/microliter	0 cm^2/μL used in Fig. 2-6.
-	Nodal inner to outer volume ratio	1	
**axon properties**			
κ	Scaled internodal conductance	0.3 mS/cm^2	Fig. S1*a* uses 0. Figs. 5 and S6 both use 4/5, 5/4 and 6/4 of this value, only for internodes 5-6 and 6-7.
Q	Damaged node number	6	Fig. 4*b* and S3*b* also have Q±1 nodes damaged
N	Number of nodes in model axon	10	1 in Fig. S1*a*, several values in Fig. S1*c*.
**simulation parameters**			
t_{LS}	Instant at which CLS is applied to damaged node(s)	0 ms	Average over several equidistant values in Fig. 3*a*
t_{κ}	Instant at which nodes are connected together	100 ms	
t_{stim}	Instant at which the stimulation starts	300 ms	
ΔV_{stim}	Amplitude of each stimulation applied at node 1	25 mV	Other values tested in Fig. S1*a*.
f_{max}	Maximum 1:1 propagation frequency	85.1 Hz	
f_{stim}	Stimulation frequency	-	$0 < f_{\mathrm{stim}} < f_{\mathrm{max}}$
**VP metric parameters**			
q	weight parameter	0.2 ms^{-1}	Various values tested in Fig. S5*a*
-	Duration of the stimulus (and of comparison)	500 ms	Various values tested in Fig. S5*b*
