# Supplementary material for: Stimulation-induced ectopicity and propagation windows in model damaged axons
Source: J Comput Neurosci. 2014 Aug 12;37(3):523–31. doi: 10.1007/s10827-014-0521-9 (PMC4224747; doi:10.1007/s10827-014-0521-9)
Supplement: Supplementary file 2 — (PDF 1.63 kb) [file 10827_2014_521_MOESM2_ESM.pdf]

Online Resource 2

Lachance M, Longtin A, Morris CE, Yu N, Joós B.

1. Additional Methods

Intact nodes of Ranvier. Each of the N = 10 nodes of Ranvier was modeled as an isopotential compartment with membrane voltage V_i given by:

$$-C\frac{\mathrm{d}V_i}{\mathrm{d}t} = I_{\mathrm{Na},i} + I_{\mathrm{K},i} + I_{\mathrm{pump},i} + I_{\mathrm{Na,leak},i} + I_{\mathrm{K,leak},i} + I_{\mathrm{leak},i} + \kappa(V_{i+1} + V_{i-1} - 2V_i) \quad \text{[S1]}$$

where i = 1, …, N is the node number. The last term on the RHS is the inter-compartment current and uses $V_0 = V_1$ and $V_{N+1} = V_N$ boundary conditions. The six first terms are transmembrane currents: the two currents (Na^+ and K^+) controlled by voltage-gated channels (VGC), the currents mediated by the population of Na/K pumps, and three ohmic leak currents (sodium, potassium and unspecific). We neglect any possible internodal sodium (Zeng *et al* 2008), although our results could extend to unmyelinated axon with sodium clusters (Zeng *et al* 2009).

VGC currents. The voltage-gated sodium and potassium currents were modeled using the equations of the Hodgkin-Huxley (HH) model (1952), with the usual kinetics for the Na- and K-gating:

$$I_{\mathrm{Na},i} = g_{\mathrm{Na}} m_i^3 h_i (V_i - E_{\mathrm{Na},i}) \quad \text{[S2]}$$

$$I_{\mathrm{K},i} = g_{\mathrm{K}} n_i^4 (V_i - E_{\mathrm{K},i}) \quad \text{[S3]}$$

where m_i and h_i model the activation and inactivation (availability) of the Nav channels while n_i is the Kv channel gating variable. However, in this work, the HH model is augmented with time-varying concentration-dependent Nernst potentials E_{Na} and E_{K} given by

$$E_{\mathrm{X},i} = \frac{kT}{e}\ln\frac{[\mathrm{X}]_{\mathrm{o},i}}{[\mathrm{X}]_{\mathrm{i},i}} \qquad \mathrm{X} = \{\mathrm{Na},\mathrm{K}\} \quad \text{[S4]}$$

where the indexes o and i denote outer and inner concentrations respectively. Time-dependence of the concentrations is described below. In the HH model, the three gating variables evolve in time according to

$$\frac{\mathrm{d}x_i}{\mathrm{d}t} = \alpha_{x,i}(1 - x_i) - \beta_{x,i} x_i \qquad x_i = \{m_i, h_i, n_i\} \quad \text{[S5]}$$

The forward (α) and backward (β) rate functions are considered to be functions of the voltage V_i only that can be found in (Hodgkin & Huxley 1952):

$$\begin{aligned} &\alpha_m = \tfrac{0.1(V+40)}{1-\exp(-4-0.1V)} && \beta_m = 4\exp(\tfrac{-V-65}{18}) \\ &\alpha_h = 0.07\exp(\tfrac{-V-65}{20}) && \beta_h = \tfrac{1}{1+\exp(-3.5-0.1V)} \\ &\alpha_n = \tfrac{0.01(V+55)}{1-\exp(-5.5-0.1V)} && \beta_n = 0.125\exp(\tfrac{-V-65}{80}) \end{aligned} \quad \text{[S6]}$$

where the indexes i = node number were dropped, here and in all following equations, to ease notation and to avoid confusion with i = inner. Previous work (Boucher *et al* 2012) argued that HH kinetics approximates results obtained with more evolved models (eg, Taddese & Bean 2002) in which the kinetic coupling of activation and inactivation (availability) in Nav channels is represented using a many-state allosteric Markov model. The HH model, however, presents the advantage of using much less computational time.

Pump currents and concentration time dependence. Even though the activity of Na/K pumps shows a small dependence on voltage, in normal conditions it depends mostly on the concentration of the substrate (Läuger 1991). Therefore, we model the pump current at each node using the Michaelis-Menten enzyme kinetics model, as was also done in (Kager *et al* 2000):

$$I_{\mathrm{pump}} = I_{\mathrm{max}}\left(\frac{[\mathrm{K}^+]_{\mathrm{o}}}{[\mathrm{K}^+]_{\mathrm{o}} + K_{\mathrm{M,K}}}\right)^2\left(\frac{[\mathrm{Na}^+]_{\mathrm{i}}}{[\mathrm{Na}^+]_{\mathrm{i}} + K_{\mathrm{M,Na}}}\right)^3 \quad \text{[S7]}$$

where the exponents show up because the pump exchanges three inner Na^+ ions for each two outer K^+ ions.

The point of introducing pump currents is to investigate time-dependent ionic concentration changes. Na^+ and K^+ concentrations at each node evolve according to

$$\frac{\mathrm{d}[\mathrm{X}]_{\mathrm{o}}}{\mathrm{d}t} = -\frac{\mathrm{d}[\mathrm{X}]_{\mathrm{i}}}{\mathrm{d}t} = \left[I_{\mathrm{X}} + I_{\mathrm{X,pump}} + I_{\mathrm{X,leak}}\right]\frac{r}{F}$$

$$\mathrm{X} = \{\mathrm{Na},\mathrm{K}\} \quad \text{[S8]}$$

where $I_{\mathrm{Na,pump}} = 3I_{\mathrm{pump}}$, $I_{\mathrm{K,pump}} = -2I_{\mathrm{pump}}$, I_{X} is the VGC current given by Eq. S2 or S3, $F = N_{\mathrm{A}}e$ = 96472 C/mol is the Faraday constant, r = 20 cm^2/μL is the surface to volume ratio of the node of Ranvier and the inner and outer volumes are considered equal for simplicity. Note that 20 is a rather high value for r, chosen to shorten computation time without

affecting phenomena qualitatively : a cylinder of length L and radius R = 5 μm would have r = 4 cm^2/μL and a real axon, where ions can effectively diffuse from a volume longer than the nodal length L, would have an even smaller r value. Using a more realistic value, phenomena reported in Figs 1, S3 and S8 would occur over a time scale about one order of magnitude larger.

Concentration changes given by Eq. S8, in turn, affect the Nernst potential that appear in Eq. S2 and S3. Since these concentration changes are orders of magnitude slower than HH kinetics, they are neglected in some simulations. We did so by setting $r = 0$ in Eq. S8, *not* by returning to a standard HH model with $I_{pump} = 0$ and $I_{X,leak} = 0$. This ensures that constant concentrations are a limiting behavior of our model, not a feature of an entirely different model; it also promotes comparability of the results.

Leak currents. The sixth term in Eq. S1 is the regular non-specific leak current of the HH model, given by

$$I_{leak} = g_{leak}(V - E_{leak}) \qquad \text{[S9]}$$

However, when pumps are added in the model, *specific leak currents* $I_{X,leak}$ are also required: indeed, the total Na^+ and K^+ currents, found between square brackets in Eq. S8, need to both be zero when the node is at rest. Since the pump current is never zero and since there is a window current involving I_{Na} and I_K even at rest potential, specific leak currents are needed to balance total Na^+ and total K^+ currents separately (Boucher *et al* 2012). They are given by

$$I_{X,leak} = g_{X,leak}(V - E_X) \qquad X = \{Na, K\} \qquad \text{[S10]}$$

All equations were solved with RK4 method with variable timestep, implemented on a PC using code in C language. The variable timestep was adjusted as suggested in (Press *et al* 1992): the 5th order term was monitored and kept just below a 4×10^{-12} target. This term was then added to the 4th order (RK4) result. The raw code is made available as Online Resource 3.

Damaged node(s) of Ranvier. In our computational model, irreversible CLS of activation and inactivation of the Nav channels is readily modeled by shifting the voltage dependence of α_m, α_h, β_m and β_h by an amount LS (i.e. replacing V by $V + LS$ in Eq. S6, in the damaged node(s) only).

Individual channels are not modeled explicitly; rather, we assume all channels in the damaged ($i = 6 \equiv Q$) node of Ranvier are left-shifted by the same LS value. In some figures, nodes $Q\pm1$ were also damaged, with the same LS value as for node Q. We do not consider situations where a distribution of LS values occurs at a same node, even though we expect our results to be easily extended to those situations.

Parameter selection. Unless stated otherwise, all parameters in the above equations take the values listed in Online Resource 1. Of those, the classical parameters (C, g_{Na}, g_K) were chosen identical to the ones in (Ochab-Marcinek *et al* 2009). The three parameters $g_{Na,leak}$, $g_{K,leak}$ and I_{max} needed to be chosen to cancel the total Na^+ current and the total K^+ currents in the resting state. These two constraints for three unknowns left one degree of freedom. The last constraint that we considered is qualitative: the node should respond readily to a stimulation current which is a small fraction of the total Na^+ current observed during an AP. This consideration led us to select a reasonably small I_{max}. Finally, three remaining parameters (κ, N and the stimulation amplitude ΔV_{stim}, to be defined) will now be discussed in greater detail.

Simulations with a damaged axon will be compared with identical simulations on an intact (control) axon. This intact axon should behave normally: each time it is stimulated, it should fire an AP and each AP fired should be propagated faithfully in all nodes. Also, behavior should be independent of N, the total number of nodes. The last three parameters, κ, N and ΔV_{stim}, were chosen to ensure this expected behavior (Fig. S1).

Stimulation at the initial node of the axon. To allow for the study of both periodic and aperiodic spike trains, node 1 has to be stimulated with timed delta functions, each causing an instantaneous ΔV_{stim} discontinuity in V_1. To select an appropriate value for ΔV_{stim}, we fed periodic stimulation of various heights ΔV_{stim} and frequencies f_{stim}, using $r = 0$ and $\kappa = 0$, and kept track of the ratio of firing frequency to f_{stim}. Fig. S1*a* shows (in green) the range where this ratio is 1:1 and shows the value selected, ΔV_{stim} = 25 mV. When $r > 0$ is restored, the system behaves normally for a prolonged stimulation period (Fig. S1*b*). When propagation is restored ($\kappa > 0$), the limit of the 1:1 zone slightly shifts towards lower frequencies (see "stim failure" limit on Fig. S1*c*).

Outside the green range on Fig. S1*a*, too large ΔV_{stim} caused period doubling while too large stimulation frequencies caused partial response, with or without phase locking (other than 1:1). Interestingly, partial responses involved a Farey sequence of phase locked zones, surrounded by zones of aperiodic behavior, much like what Feingold *et al* (1988) obtained in an externally driven van der Pol oscillator (see also Aihara *et al* 1986; Kaplan *et al* 1996). Also, the green zone boundaries were slightly dependent on E_{Na} and E_K, as shown by the curves in Fig. S1*a*. The tested values of E_{Na} and E_K are typical of the depletion that occurs during a prolonged (300 ms) stimulation (red and green curves on Fig. S1*b*).

Propagation along the axon and length of the axon. In Eq. S1, the saltatory propagation along the computational axon was modeled by considering each internode as a single ohmic conductance κ linking two consecutive nodes of Ranvier. This is equivalent to assuming that the myelinated internodes have negligible capacitance. Consequently, their RC charging time constant is so small compared to the node

of Ranvier's that the nodes can be assumed to directly charge one another. This approach was previously used in (Ochab-Marcinek *et al* 2009) and in a small portion of (Boucher *et al* 2012), although only constant current stimulation was used.

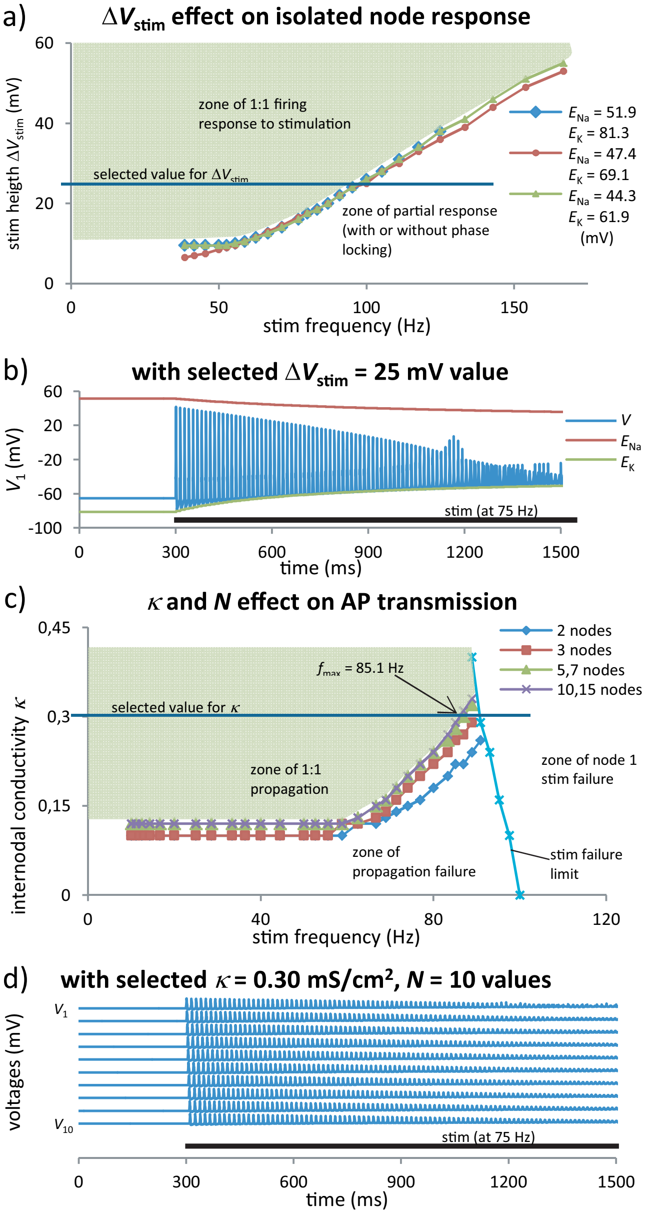

Fig. S1. Parameter tuning of the intact (control) system. (a) In green : range of stimulation strengths (ΔV_{stim}) and stimulation (stim) frequencies for which the isolated first node fires each time it is stimulated. The limit of the green zone (bounded by the curves) is recomputed for three fixed E_{Na} and E_K values ($r = 0$), with $\kappa = 0$. Value selected and used throughout this article : $\Delta V_{stim} = 25$ mV. (b) Typical response of isolated node ($\kappa = 0$) to prolonged periodic stimulation (denoted by black bar), once $r = 20$ $cm^2/\mu L$ is restored ($\Delta V_{stim} = 25$ mV). Note that this r value is chosen large to promote rapid depletion of the Nernst potentials. (c) In green : range of internode conductances (κ) and stim frequencies for which APs fired in node 1 are faithfully propagated to all N nodes. Limit of green zone is recomputed for various axon lengths (N), with $r = 0$. Values selected and used throughout this article: $N = 10$ nodes and $\kappa = 0.30$ mS/cm^2. Note that the control system behaves normally for all stim frequencies <85.1 Hz. (d) Typical response to stimulation of the 10-node intact axon, once $r = 20$ $cm^2/\mu L$ is restored (stim as in (b), $\kappa = 0.30$ mS/cm^2). The top node (1) is the one being stimulated.

To select an appropriate value for κ, we fed periodic stimulation ($\Delta V_{stim} = 25$ mV) of various f_{stim}, using $r = 0$, to axons having a range of κ values, and kept track of the ratio f_N/f_1 of the firing frequencies of node N and node 1. Fig. S1*c* shows (in green) the range where this ratio is 1:1 and the value selected, $\kappa = 0.3$ mS/cm^2. (We separately tested that κ was still low enough to prevent subthreshold oscillations to be propagated.) For short axons, the limit of the green zone on Fig. S1*c* was found to be N-dependent, but not above $N \approx 10$, the value we have thus chosen. At the right of the "stim failure" line, $f_1 < f_{stim}$ (zone of "stim failure") while at its left, despite the fact that $f_1 = f_{stim}$, there is a zone where $f_{10} = f_1$ (green zone) and another where $f_{10} < f_1$ (zone of "propagation failure"). Therefore, at $\kappa = 0.3$ mS/cm^2, propagation failure can be distinguished from stimulation failure. As for stim failure, propagation failure involves a Farey sequence of phase locking zones. When $r > 0$ is restored, the system behaves normally for a prolonged stimulation period (Fig. S1*d*). It does so for all firing frequencies ≤ 85.1 Hz.

2. Additional Results

Onset of ectopic activity. Fig. S2 shows the limit between quiescent and ectopic behaviors as a function of E_{Na} and E_K. Diminishing E_K reduces the K^+ driving force and increases the rest potential, bringing it closer to firing threshold. Diminishing E_{Na} partially inverts that effect, but occurs too slowly to prevent the onset of ectopicity. Note that once ectopicity is triggered, further decline of E_K increases f_Q (see also Fig. S8).

Fig. S3 shows how the propagation of normal APs (i.e. those caused by a stimulation in node 1) can gradually diminish E_{Na} and E_K until the boundary shown in Fig. S2 is crossed. The top panel of Fig. S3*a* is a repetition of Fig. 1*b* for comparison and the top panel of Fig. S3*b* is the same obtained with nodes 5-7 damaged, while the middle and lower panels are, respectively, the values of E_K and E_{Na} at the time of the next stimulation following the onset of ectopic activity. Note how ectopic activity is triggered when a limit value of E_K is reached, whatever the value E_{Na} reached at the same time (this is mostly obvious on low f_{stim} curves).

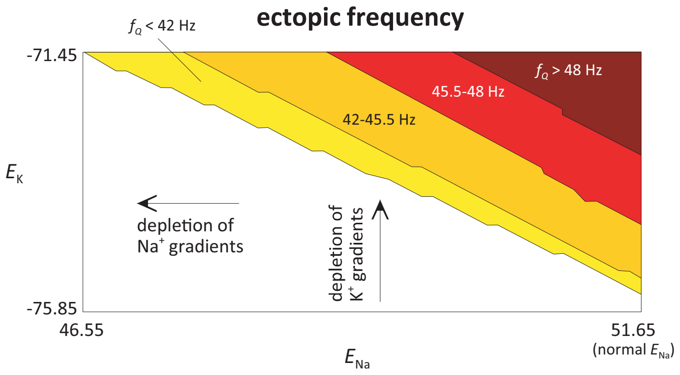

Fig. S2. Ectopic frequency as a function of E_{Na} and E_K, drawn for $LS = 3$ mV, $r = 0$, $t_{LS} = t_\kappa = 0$ and no stimulation. White zone = not ectopic. Note that diminishing E_K brings the system into ectopicity, while diminishing E_{Na} does the opposite.

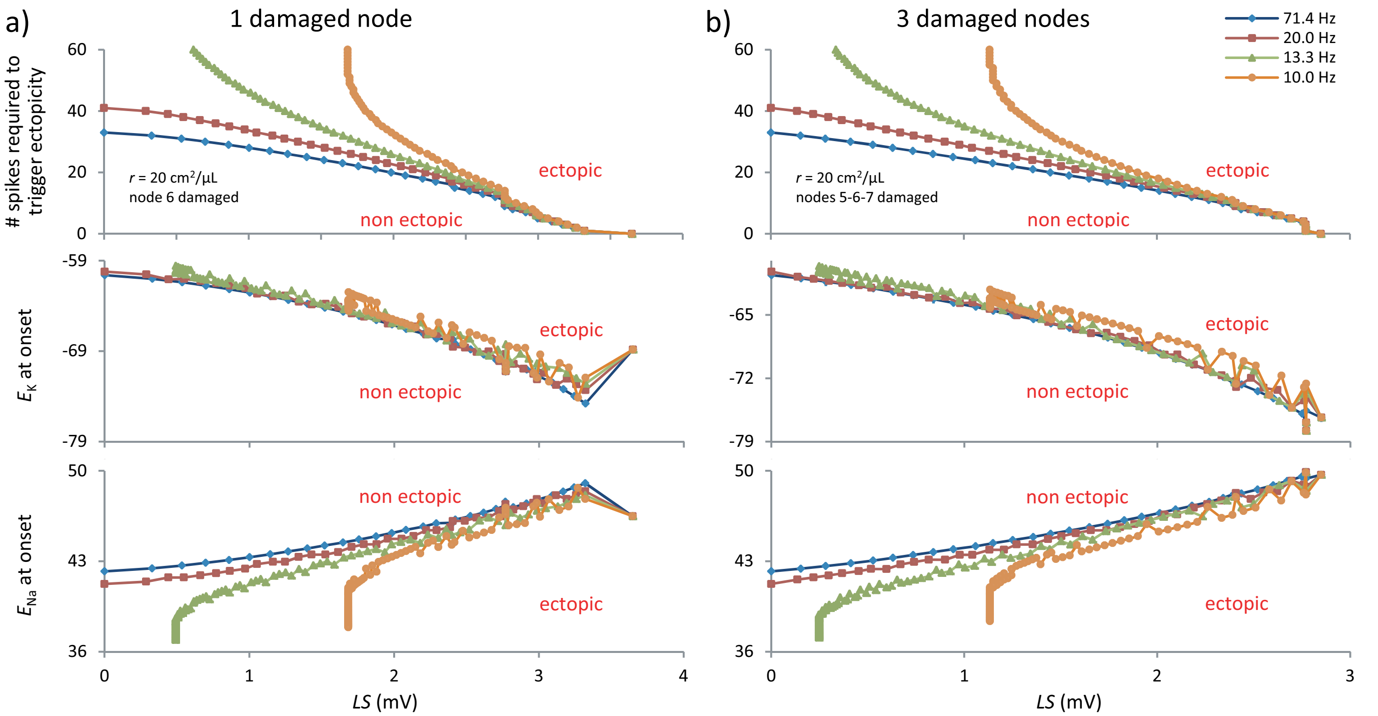

Fig. S3. Triggered ectopicity. (a) First panel: repetition of Fig 1b. Middle and lower panels: corresponding E_K and E_{Na} at the time of the next stimulation following the onset of ectopic activity. (b) Same as (a), with nodes 5-7 damaged. Note the change in horizontal scale.

Evaluation of the settling time in Fig. 3*a*. To draw Fig. 3*a*, the following procedure was followed:

1) Delays between the nth output spike in control and damaged axons were computed.

2) We tested if those delays converged towards a constant (potentially non zero) value (a small fluctuation was tolerated). If not, the settling time is infinite (longer than the simulation duration) and procedure stops. Otherwise, 1:1 phase locking is considered detected.

3) If phase locking is detected, we found the instant t_{lock} when consecutive delays started differing by less than 0.5 ms (we ensured that this difference didn't re-increase later by conducting the search with decreasing t). Once this instant was located between two consecutive spikes, we linearly interpolated the precise t_{lock}.

4) The settling time was then equal to $t_{lock} - t_{first}$, where t_{first} is the instant when the first AP reaches node 10 in the control system.

5) Finally, the result of step 4 was found to be dependent on the phase of the ectopic node at the instant when stimulation begins (remember: since node 6 is ectopic, it already fires at frequency f_Q before stimulation begins). To eliminate the influence of this factor, steps 1-4 were repeated 5 times, using equidistant phases obtained by changing t_{LS}. The five results were then averaged to yield a point of Fig. 3*a*.

VP metric (output infidelity). The Victor-Purpura (VP) metric (1996) allows to compare two spike trains (S_a and S_b on Fig. S4) by evaluating the minimal series of elementary steps required to transform one of these trains into the other. Two elementary steps are possible, a "cost" being associated to each: adding/removing a spike (cost = 1) or shifting a spike by Δt (cost = $q\Delta t$, where q is an arbitrary parameter). All possible ways to convert S_a into S_b using these steps are computed (Fig. S4 shows a single one of them) and the *minimal* cost is considered a measure of the difference between S_a and S_b, their "VP distance".

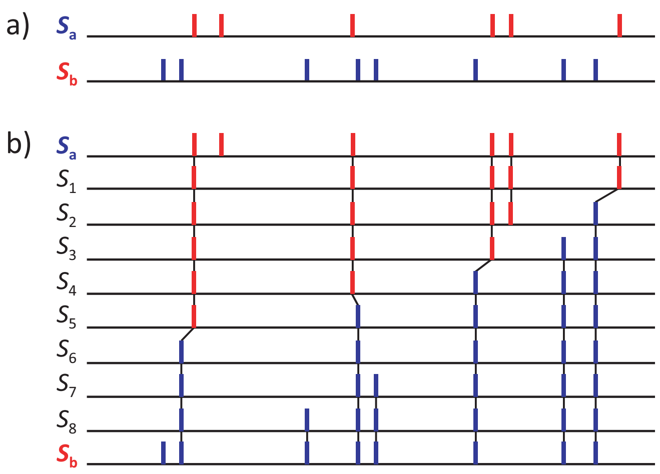

Fig. S4. VP metric. (a) Two spike trains to be compared. (b) An example series of steps transforming S_a into S_b, as involved in the VP metric that we use to measure infidelity. Modified from (9).

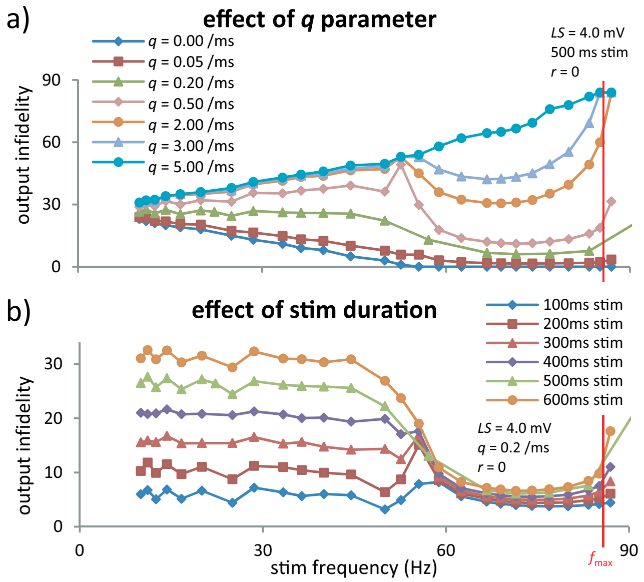

Fig. S5. Infidelity parameters (q and total stimulus duration) have no qualitative effect on results. (a) LS = 4 mV curve from Fig. 4*a* recomputed for various q values. Note that all curves $q < 5\ \mathrm{ms}^{-1}$ show the propagation window. (b) LS = 4 mV curve from Fig. 4*a* recomputed for a range of stimulus intervals. Note that, as the stim duration is increased, settling time represents a smaller proportion of the comparison interval.

Using the VP metric to evaluate output infidelity takes into account both settling time (during which a number of spikes are added) and phase shifts, thus producing a reasonable measure of output infidelity. In Fig. S5*a*, we show results for a range of q values.

As shown in (Victor & Purpura 1996), this "VP distance" matches all the mathematical conditions to qualify as a metric. Therefore, if one imagines an abstract space where each possible spike train is a point, the cost obtained can be seen as a distance between these two points.

Effect of parameters on output infidelity. Fig. 4 used q = 0.2 ms^{-1} and 500 ms of stimulation / comparison time. Fig. S5 shows that one of our main results, namely the existence of a "propagation window", is independent of these two choices: except for very large q values, a propagation window is always observed (Fig. S5*a*). Also, increasing the duration of the stimulation (during which the output trains are compared), simply reduces the relative importance of the settling time, thus deepening the minimum band corresponding to the propagation window (Fig. S5*b*).

Note that the q = 0 curve on Fig. S5*a* shows zero output infidelity in the propagation window, meaning identical spike count. The non zero infidelity seen for identical f_{stim} on Fig. 4*a* (where $q > 0$) is therefore due only to timing differences, which are in turn due to the settling time and to the phase shift that we described in Fig. 3.

Effect of local conductance. While Fig. 5 shows the effect of local increase or decrease in radius when LS = 4 mV, Fig. S6 shows the same for other values of LS.

Generation of jitter and noise. To produce each of the jittered inputs, we first generated a deterministic periodic spike train. Then, for each of the spikes in this input train, we used a random number generator to select a "displacement" from a Gaussian distribution of fixed standard deviation "*stdev*" (see legend on Fig. 6*a*). A total of 50 spike trains were thus obtained and saved, each with random displacements selected from the same Gaussian distribution. Each of those 50 input trains was then fed both to the control and damaged axons, producing each time two outputs that were compared using the usual VP metric to measure output infidelity. (This way, compared outputs are always affected by the same random effects.) Finally, the 50 infidelity values were averaged to produce one point on Fig. 6*a*.

A similar procedure was used to produce Fig. 6*b*: for each *node*, 50 lists of random numbers were selected from a Gaussian distribution with unit standard deviation (i.e. they are the increments of a Wiener process, ΔW_n). At each time step n, we added $A\sqrt{h}\Delta W_n$ to the voltage of the node, where ΔW_n is the nth number from that node's list, A is the noise amplitude (see legend on Fig. 6*b*) and h is the integration time step. Note that when noise was present, we turned off the adaptative time step and used h = 0.001 ms. As

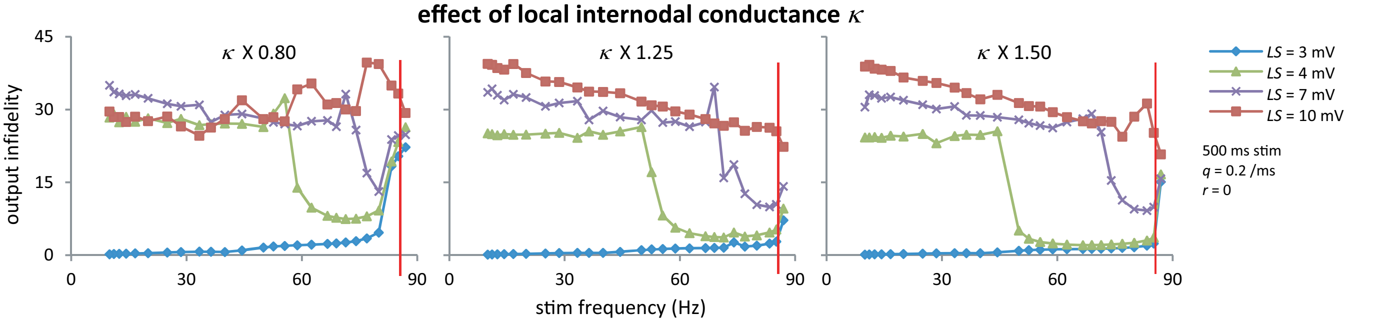

Fig. S6. Local increase/decrease of internodal conductance, as expected if the axon swells/shrinks in reaction to injury, reduces/increases infidelity. Figure is the same as Fig. 5, except that LS takes different values.

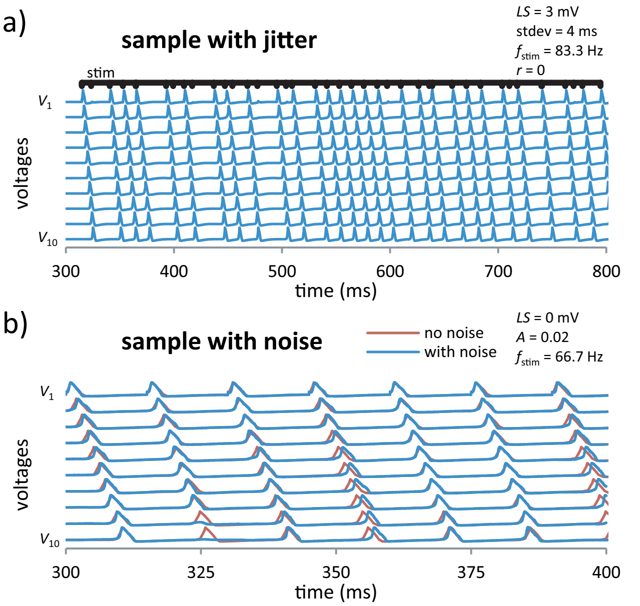

Fig. S7. Sample time series, with jitter or noise. (a) This relatively important jitter (stdev = 33% of stimulation period) is sufficient to cause stimulation failure (compare times of stimulation, indicated above node 1, with actual firing times of node 1 firings). (b) This noise (A = 0.02) is sufficient to cause propagation failure, as can be seen by comparing curves with/without noise (second spike from the left fails between nodes 8 and 9).

in Fig. 6*a*, the same lists were used both for the intact and damaged system. The outputs were compared using the VP metric and the 50 output infidelities were averaged to produce a point on Fig. 6*b*.

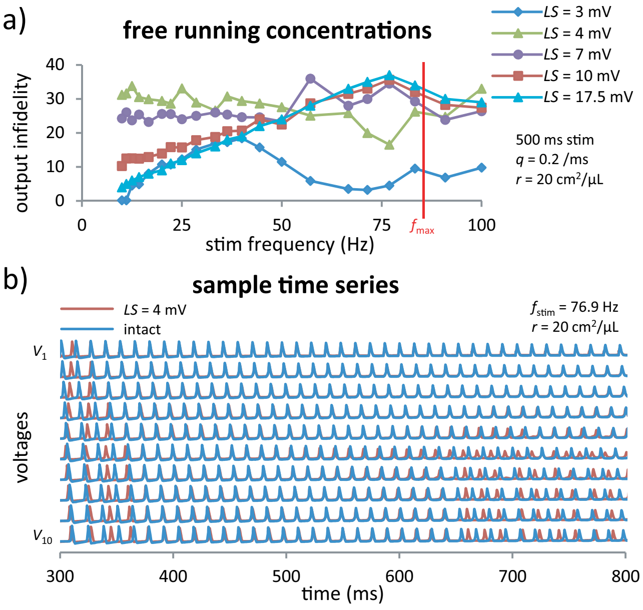

Fig. S8. Gradient depletion renders the propagation window transient. (a) Same as Fig. 4*a*, but with r = 20 cm^2/µL. Note the high infidelity for LS = 3 mV, due to triggered ectopicity (see Figs. 1 and S3). (b) Sample time series showing a settling time of 60-70 ms, followed by about 300 ms of phase locking and then by a loss of phase locking. If infidelity were evaluated for 350 ms of stimulus instead of 500 ms, it would be smaller here.

To help visualize the relative importance of jitter with respect to quasiperiod or the importance of noise relative to voltage excursions, Fig. S7 shows an example time series of each.

Output infidelity with free running ionic concentrations. When r = 20 cm^2/µL, pumps cannot speed up enough to prevent E_{Na} and E_K from declining. At first, the Nernst potentials are nearly unchanged. Therefore, mildly damaged axons, for which the "settling time" is the shortest, have no trouble phase locking 1:1. However, their ion gradients eventually deplete, f_Q increases, the 1:1 phase locking condition is lost. Therefore, for the total stimulus duration (500 ms), the propagation window is shallower. Fig. S8 shows this, both with a time series and with an output infidelity diagram.

References

Aihara K., Numagiri T., Matsumoto G., and Kotani M., *Phys. Lett. A* 116, 313 (1986)

Boucher P.A., Joós B., Morris C.E. (2012) Coupled left-shift of Nav channels: modeling the Na+-loading and dysfunctional excitability of damaged axons. *Journal of computational neuroscience* 33(2):301-319.

Feingold M., Gonzalez D.L., Piro O., Viturro H. (1988) Phase locking, period doubling, and chaotic phenomena in externally driven excitable systems.*Physical Review A* 37(10):4060-4063.

Hodgkin A.L., Huxley,A.F. (1952) A quantitative description of membrane current and its application to conduction and excitation in nerve. *The Journal of physiology* 117(4):500.

Kager H., Wadman W.J., Somjen G.G. (2000) Simulated seizures and spreading depression in a neuron model incorporating interstitial space and ion concentrations. *Journal of neurophysiology* 84(1):495-512.

Kaplan D.T., Clay J.R., Manning T., Glass L., Guevara M.R., Shrier A., Subthreshold dynamics in periodically stimulated squid giant axons. *Phys. Rev. Lett.* 76, 4074 (1996).

Läuger P. (1991) *Electrogenic ion pumps* (Vol. 5). Sunderland: Sinauer Associates.

Ochab-Marcinek A., Schmid G., Goychuk I., Hänggi P. (2009) Noise-assisted spike propagation in myelinated neurons. *Physical Review E 79*(1):011904.

Press W.H., Teukolsky S.A., Vetterling W.T., Flannery B.P. (1992) Numerical Recipes in C: The Art of Scientific Computing, Cambridge.

Taddese, A., & Bean, B. P. (2002). Subthreshold sodium current from rapidly inactivating sodium channels drives spontaneous firing of tuberomammillary neurons. *Neuron* 33(4):587-600.

Victor J.D., Purpura K.P. (1996) Nature and precision of temporal coding in visual cortex: a metric-space analysis. *Journal of Neurophysiology* 76(2):1310-1326.

Zeng S. and Jung P., *Phys Rev E* 78, 061916 (2008).

Zeng S. and Tang Y., *Phys Rev E* 80, 021917 (2009).
